# Improving on-treatment risk stratification of cancer patients with refined response classification and integration of circulating tumor DNA kinetics

**DOI:** 10.1186/s12916-022-02463-5

**Published:** 2022-08-23

**Authors:** Jiawei Lv, Chenfei Wu, Junyan Li, Foping Chen, Shiwei He, Qingmei He, Guanqun Zhou, Jun Ma, Ying Sun, Denghui Wei, Li Lin

**Affiliations:** 1 Department of Radiation Oncology, Sun Yat-sen University Cancer Center, the State Key Laboratory of Oncology in South China, Collaborative Innovation Center for Cancer Medicine, Guangdong Key Laboratory of Nasopharyngeal Carcinoma Diagnosis and Therapy, Center for Precision Medicine of Sun Yat-sen University, Guangzhou, 510060 People’s Republic of China; 2 State Key Laboratory of Oncology in Southern China, Collaborative Innovation Center for Cancer Medicine, Sun Yat-sen University Cancer Center, Guangzhou, China

**Keywords:** Liquid biopsy, Circulating tumor DNA, RECIST, Risk-adapted personalized therapy, Nasopharyngeal carcinoma

## Abstract

**Background:**

Significant intertumoral heterogeneity exists as antitumor treatment is introduced. Heterogeneous therapeutic responses are conventionally evaluated by imaging examinations based on Response Evaluation Criteria in Solid Tumors (RECIST); nevertheless, there are increasing recognitions that they do not fully capture patient clinical benefits. Currently, there is a paucity of data regarding the clinical implication of biological responses assessed by liquid biopsy of on-treatment circulating tumor DNA (ctDNA). Here, we investigated whether biological response evaluated by ctDNA kinetics added critical information to the RECIST, and whether integrating on-treatment biological response information refined risk stratification of cancer patients.

**Methods:**

In this population-based cohort study, we included 821 patients with Epstein-Barr virus (EBV)-associated nasopharynx of head and neck cancer (NPC) receiving sequential neoadjuvant chemotherapy (NAC) and chemoradiotherapy (CRT), who had pretreatment and on-treatment cfEBV DNA and magnetic resonance imaging (MRI) surveillance. Biological responses evaluated by cfEBV DNA were profiled and compared with conventional MRI-based RECIST evaluation. The inverse probability weighting (IPW)-adjusted survival analysis was performed for major survival endpoints. The Cox proportional hazard regression [CpH]-based model was developed to predict the on-treatment ctDNA-based individualized survival.

**Results:**

Of 821 patients, 71.4% achieved complete biological response (cBR) upon NAC completion. RECIST-based response evaluations had 25.3% discordance with ctDNA-based evaluations. IPW-adjusted survival analysis revealed that cfEBV DNA_post-NAC_ was a preferential prognosticator for all endpoints, especially for distant metastasis. In contrast, radiological response was more preferentially associated with locoregional recurrence. Intriguingly, cfEBV DNA_post-NAC_ further stratified RECIST-responsive and non-responsive patients; RECIST-based non-responsive patients with cBR still derived substantial clinical benefits. Moreover, detectable cfEBV DNA_post-NAC_ had 83.6% prediction sensitivity for detectable post-treatment ctDNA, which conferred early determination of treatment benefits. Finally, we established individualized risk prediction models and demonstrated that introducing on-treatment ctDNA significantly refined risk stratification.

**Conclusions:**

Our study helps advance the implementation of ctDNA-based testing in therapeutic response evaluation for a refined risk stratification. The dynamic and refined risk profiling would tailor future liquid biopsy-based risk-adapted personalized therapy.

**Supplementary Information:**

The online version contains supplementary material available at 10.1186/s12916-022-02463-5.

## Background

Cancer patients demonstrate significant intertumoral heterogeneity [1, 2]. Such biological variations contribute to dramatically different outcomes. Over the past decades, increasing efforts have been made in unraveling this heterogeneity through histological, radiographic, and molecular dissection [3–5]. However, published evidence has largely focused on pretreatment parameters and generally does not consider the dynamic response information, partly due to the difficulty in obtaining serial tumor samples. Nevertheless, there is substantial evidence that tumor dynamic response to systemic therapy harbors critical clinical implications, which demonstrates significant heterogeneity even across patients sharing identical pretreatment characteristics [6–8]. For example, a subset of high-risk patients can achieve exceptional responses and be cured with frontline therapy, while other patients with identical pretreatment risk factors do not respond well to frontline therapy and develop disease recurrence soon after treatment completion.

Clinically, tumor responses are routinely evaluated by conventional imaging examinations based on the Response Evaluation Criteria in Solid Tumors (RECIST) or pathological/biopsy materials. However, these conventional approaches do not fully capture the tumor biological features or the dynamics of clinical benefits over time. For example, over 30% of patients with lung cancer who had stable disease (SD) at the first imaging scanning can ultimately achieve durable clinical benefits [9, 10]. On the other hand, repeated tumor biopsies are not clinically feasible and may not capture timely information during the treatment course. On this note, there is a considerable unmet need for early response assessment methods that can reflect tumor biology in a more accurate way; meanwhile, identify patients with long-term tumor control in a timely manner.

Liquid biopsy of circulating tumor DNA (ctDNA) in the peripheral blood provides noninvasive access to cancer-specific genomes and biology [11]. Accumulating evidence suggests that ctDNA quantification and on-treatment changes provide informative information on therapeutic responses, tumor biology, and risk stratification [12, 13]. Despite the promising data, their clinical utility to date has remained limited in various cancers, given the small study cohorts and heterogeneous treatment modalities, especially from the aspects of response assessment and risk-adapted treatment guidance. Moreover, the associations between ctDNA and RECIST-based response assessment remain poorly understood.

Head and neck cancer is a heterogeneous epithelial tumor with subsets of tumors demonstrating strong associations with virus infection [14]. Nasopharyngeal carcinoma (NPC) is one of the most aggressive head and neck cancer originated from nasopharynx and is typically associated with Epstein-Barr virus (EBV) infection in patients from endemic areas [15]. Plasma circulating cell-free EBV DNA (cfEBV DNA) is a sensitive and specific biomarker for EBV-associated NPC, which consists of short DNA fragments released by NPC cells and can be detected using ultrasensitive polymerase chain reaction (PCR)-based assays [16]. In this vein, NPC represents a suitable model here for addressing the value of on-treatment ctDNA kinetics.

Here, we hypothesized that biological response evaluated by ctDNA kinetics added critical information to RECIST, and integrating on-treatment biological and radiological response information refined patient risk stratification for personalized clinical decision-making. In this investigation, we tested this hypothesis in a large-scale patient cohort with nasopharynx of head and neck cancer consistently treated with sequential neoadjuvant chemotherapy (NAC) and chemoradiotherapy (CRT), who had pretreatment and on-treatment cfEBV DNA and imaging surveillance. We demonstrated that biological response to NAC assessed by on-treatment cfEBV DNA demonstrated 25.3% discordance with RECIST-based response evaluation; moreover, they harbored important prognostic information not only complementary to but also beyond the conventional radiological responses. In addition, on-treatment cfEBV DNA kinetics conferred early determination of treatment benefits, and delayed ctDNA responses indicated unfavorable outcomes. Finally, we established risk prediction models and demonstrated that introducing on-treatment ctDNA significantly refined risk stratification of cancer patients. Our findings help advance the implementation of ctDNA-based testing in therapeutic response evaluation for a refined risk stratification. Consequently, the dynamic and refined on-treatment risk profiling would inform future risk-based therapeutic adaptation for personalized medicine in cancer patients.

## Results

### Patient cohort, clinical characteristics, and recurrence patterns

**Fig. 1:**
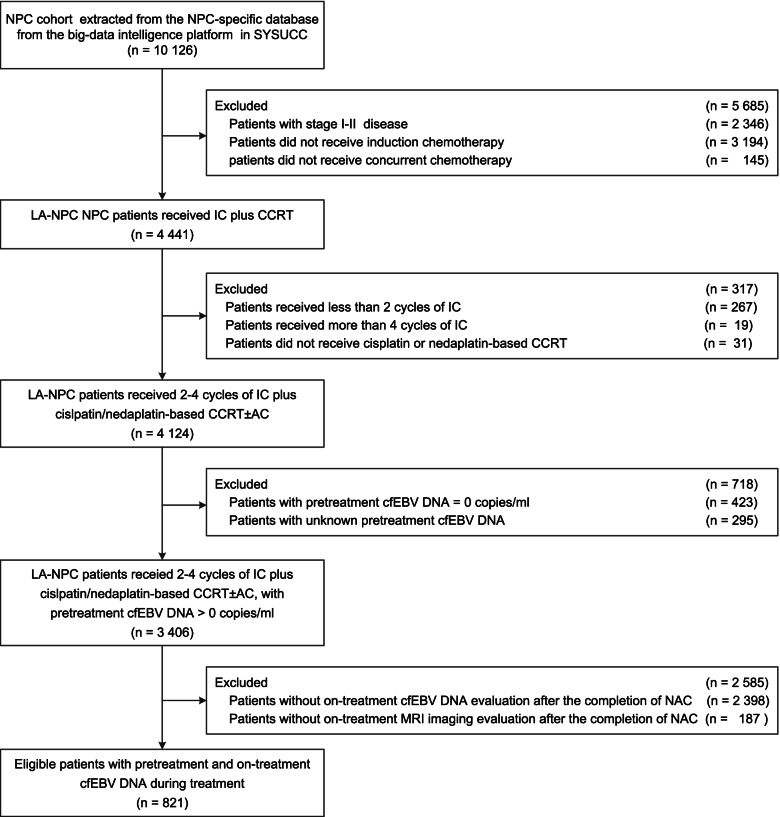
Flowchart showing the study design and patient selection process. The medical records of 10,126 patients with non-metastatic NPC were screened, and 821 patients with LA-NPC who received NAC plus concurrent CRT and had detectable pretreatment cfEBV DNA with on-treatment circulating cfEBV DNA surveillance were selected stepwise. Abbreviations: AC, adjuvant chemotherapy; CCRT, concurrent chemotherapy; cfEBV DNA, cell-free Epstein-Barr virus DNA; IC, induction chemotherapy; LA-NPC, locally advanced nasopharyngeal carcinoma; MRI, magnetic resonance imaging

We investigated 821 patients with advanced-stage EBV-associated NPC enrolled between 2009 and 2015, who consistently received cisplatin-based NAC followed by CRT. The diagram of the study population is shown in Fig. 1. The baseline clinical characteristics are presented in Table 1. Blood samples were collected at baseline and after the completion of NAC (post-NAC) and CRT (post-CRT). On-treatment imaging evaluation was conducted post-NAC using magnetic resonance imaging (MRI). The collection schema of cfEBV DNA and MRI is presented in Additional file 1: Fig. S1. The median follow-up was 64.9 months (interquartile range [IQR]: 58.1–72.5 months). We recorded 109 locoregional recurrences, 143 distant metastases, and 28 synchronous locoregional and metastatic recurrences. The 5-year rates of disease-free survival (DFS), overall survival (OS), distant metastasis-free survival (DMFS), and locoregional relapse-free survival (LRFS) were 72.5%, 82.9%, 83.1%, and 87.1%, respectively.

**Table 1:** Baseline clinical characteristics of the 821 patients with locally advanced nasopharynx of head and neck cancer

Characteristics	No. of patients (%)
Age, years	
<45	477 (58.1)
≥45	344 (41.9)
Sex	
Male	617 (75.2)
Female	204 (24.8)
Histology, WHO type^a^	
II	14 (1.7)
III	807 (98.3)
Smoking	
No	514 (62.6)
Yes	307 (37.4)
Alcohol	
No	695 (84.7)
Yes	126 (15.3)
Clinical stage^b^	
III	350 (42.6)
IV	471 (57.4)
T stage^b^	
T1	48 (5.8)
T2	71 (8.6)
T3	388 (47.3)
T4	314 (38.2)
N stage^b^	
N0	36 (4.4)
N1	311 (37.9)
N2	261 (31.8)
N3	213 (25.9)
NAC regimens	
TPF	501 (61.0)
TP	194 (23.6)
GP	54 (6.6)
PF	61 (7.4)
Others^c^	11 (1.3)
NAC cycles	
2 cycles	393 (47.9)
3 cycles	392 (47.7)
4 cycles	36 (4.4)

*Abbreviations*: *GP* Gemcitabine and cisplatin, *N* Node, *NAC* Neoadjuvant chemotherapy, *PF* Cisplatin and 5-fluorouracil, *T* Tumor, *TP* Docetaxel and cisplatin, *TPF* Docetaxel, cisplatin, and 5-fluorouracil, *WHO* World Health Organization

^a^WHO Type II refers to the differentiated non-keratinizing carcinoma; WHO Type III refers to the undifferentiated non-keratinizing carcinoma

^b^According to the 8th edition of the AJCC/UICC Staging System

^c^Others included patients with alteration of NAC regimens, for example switch from TPF to GP due to adverse events

### Biological responses to NAC and their correlations with radiological response

**Fig. 2:**
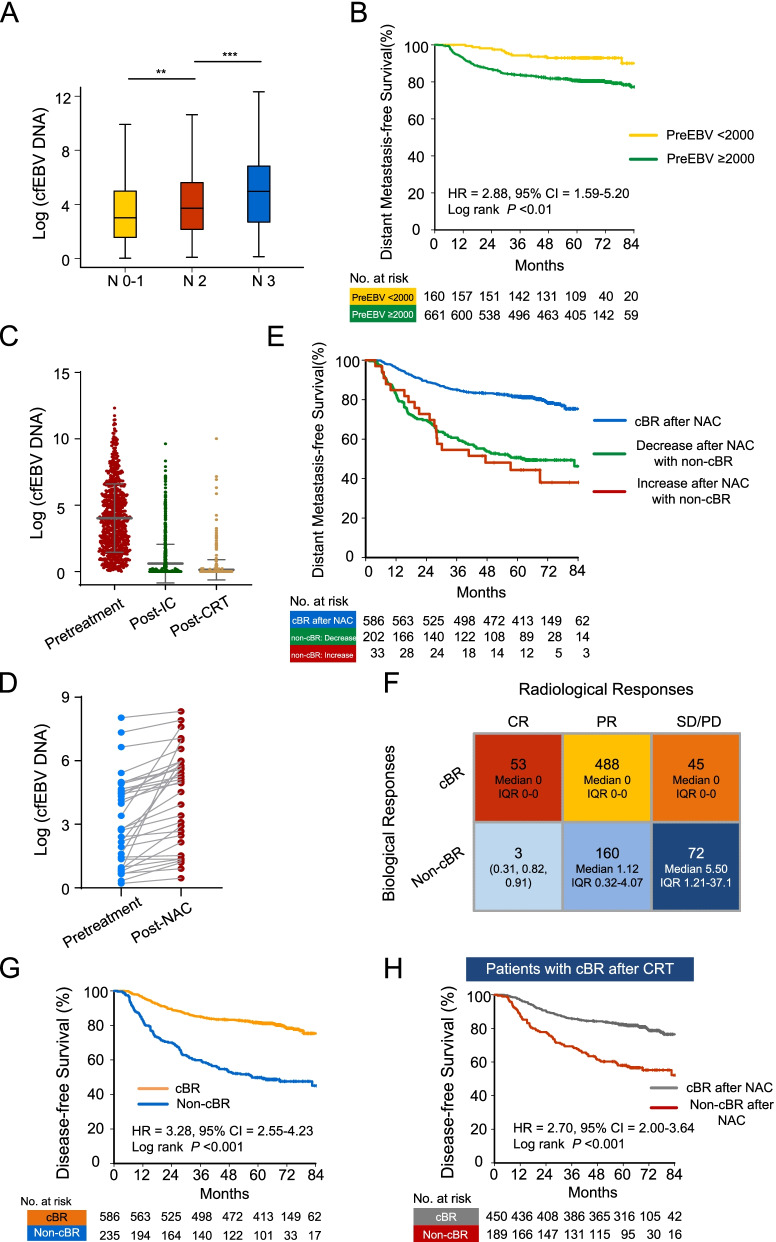
Biological responses to NAC and their correlations with radiological responses. **A** Comparison of pretreatment cfEBV DNA levels across N categories. **B** Kaplan-Meier survival plot of DMFS in patients with pretreatment cfEBV DNA ≥ 2000 copies/mL versus <2000 copies/mL. **C** Scatter plot showing circulating cfEBV DNA levels before treatment initiation, at NAC completion (post-NAC), and at CRT completion (post-CRT). **D** Changes in cfEBV DNA from baseline in patients with increased cfEBV DNA levels post-NAC (*n* = 33). **E** Kaplan-Meier survival plot of DMFS in patients with cBR post-NAC versus decreased/increased cfEBV DNA in patients with non-cBR. **F** RECIST groupings (columns) and cfEBV DNA biological responses (rows) of 821 patients with matched treatment-naïve and post-NAC surveillance data. **G** Kaplan-Meier survival plot of DFS in patients with cBR versus non-cBR post-NAC. **H** Kaplan-Meier survival plot of DFS in patients achieving cBR at the end of CRT stratified by biological responses to NAC. **A**bbreviations: cBR, complete biological response; cfEBV DNA, cell-free Epstein-Barr virus DNA; CI, confidence interval; CR, complete response; CRT, chemoradiotherapy; DFS, disease-free survival; DMFS, distant metastasis-free survival; HR, hazard ratio; IC, induction chemotherapy; N, node; NAC, neoadjuvant chemotherapy; non-cBR, non-complete biological response; PD, progression disease; PR, partial responses; PreEBV, pretreatment cfEBV DNA; SD, stable disease

All patients (*n* = 821) had detectable cfEBV DNA at baseline. The distribution of pretreatment cfEBV DNA titers (median, 12.50 × 10^3^ copies/mL; IQR, 2.96–52.50 × 10^3^ copies/mL) are shown in Additional file 1: Fig. S2A, with 661 (80.5%) patients having pretreatment cfEBV DNA higher than 2000 copies/mL. Correlation analyses revealed that pretreatment cfEBV DNA was positively associated with node (N) stage (*P* < 0.05, Wilcoxon test; Fig. 2A), but not tumor (T) stage, age, sex, and smoking status (*P* > 0.05). Additionally, in line with our previous observations [17], higher baseline cfEBV DNA load (cut-off value, 2000 copies/mL) was preferentially associated with worse survival outcomes, especially with the occurrence of distant metastasis (hazard ratio [HR] = 2.88, 95% confidence interval [CI] = 1.59–5.20, *P* < 0.01; Fig. 2B). It remained significant after correcting for clinically important covariates using the inverse probability weighting (IPW) algorithm (HR_DMFS_ = 2.51; 95% CI = 1.46–4.32; *P* < 0.01; Additional file 2: Table S1), suggesting that in addition to their well-acknowledged reflection on tumor burden, higher pretreatment cfEBV DNA levels may also be related to tumoral biological features (i.e., sensitivity to treatment and/or tumor microenvironmental heterogeneity as were referred in previously published researches [18–20]). Comparisons of the baseline covariates in the unadjusted and IPW-adjusted cohorts are shown in Table 2, demonstrating that the IPW succeeded in generating balanced distributions of covariates across subgroups.

**Table 2:** Comparison of baseline characteristics between subgroups in the unadjusted and inverse probability weighting (IPW)-adjusted cohorts

Covariates	Pre cfEBV DNA		Post-NAC MRI		Post-NAC cfEBV DNA		Response phenotypes	
	UN	IPW^a^	UN	IPW^b^	UN	IPW^b^	UN	IPW^b^
**Age**	0.51	0.59	0.58	0.83	0.19	0.85	0.14	0.59
**Sex**	0.71	0.53	0.15	0.58	0.18	0.77	0.08	0.16
**Smoking**	0.37	0.82	0.36	0.74	0.11	0.99	0.17	0.43
**Alcohol**	0.96	0.70	0.59	0.86	0.68	0.77	0.32	0.52
**Pre cfEBV DNA**	–	–	0.76	0.76	<0.01	0.99	<0.01	0.14
**T stage**	0.43	0.94	0.57	0.65	0.42	0.80	<0.01	0.23
**N stage**	<0.01	0.87	0.72	0.46	<0.01	0.94	<0.01	0.10
**NAC cycles**	0.94	0.95	0.02	0.40	0.17	0.97	0.02	0.22
**NAC regimens**	0.12	0.97	<0.01	0.29	0.27	0.87	0.03	0.09
**CCD**	0.71	0.46	0.27	0.29	0.53	0.83	0.03	0.10

*Abbreviations*: *CCD* Cumulative cisplatin doses, *cfEBV DNA* Cell-free Epstein-Barr virus DNA, *IPW* Inverse probability weighting, *MRI* Magnetic resonance imaging, *N* Node, *NAC* Neoadjuvant chemotherapy, *Pre* Pretreatment, *T* Tumor, *UN* Unadjusted

^a^The following variables were adjusted via IPW algorithm: age (<45 vs. ≥45 years), sex (male vs. female), smoking (No vs. Yes), alcohol (No vs. Yes), T stage (T1-2 vs. T3-4), N stage (N0-1 vs. N2-3), NAC regimens (TPF vs. GP vs. TP vs. PF vs. others), NAC cycles (2 cycles vs. 3 cycles vs. 4 cycles), CCD (<160vs. ≥160 mg/m^2^). Two-sided *P*-values were calculated using the chi-square test

^b^The following variables were adjusted via IPW algorithm: age (<45 vs. ≥45 years), sex (male vs. female), smoking (No vs. Yes), alcohol (No vs. Yes), pretreatment EBV DNA (<2 vs. ≥2 × 10^3^ copies/mL), T stage (T1-2 vs. T3-4), N stage (N0-1 vs. N2-3), IC regimens (TPF vs. GP vs. TP vs. PF vs. others), IC cycles (2 cycles vs. 3 cycles vs. 4 cycles), CCD (<160 vs. ≥160 mg/m^2^). Two-sided *P*-values were calculated using the chi-square test

Upon the initiation of NAC, 586 patients (71.4%) achieved complete biological response (cBR; defined as undetectable cfEBV DNA) during the NAC phase (Fig. 2C); the distributions of post-NAC cfEBV DNA titers (median, 0 copies/mL; IQR, 0–0.20 × 10^3^ copies/mL) are shown in Additional file 1: Fig. S2B. Among 235 patients with non-complete biological response post-NAC (non-cBR; defined as detectable cfEBV DNA; median, 1.55 × 10^3^ copies/mL; IQR, 0.41–7.84 × 10^3^ copies/mL), 33 (14.0%) had increased cfEBV DNA levels from baseline, which demonstrated worse prognosis (Fig. 2D, E).

Regarding the RECIST-based radiological assessment, 56 patients (6.8%) achieved complete response (CR), 648 (78.9%) patients achieved partial response (PR) during the NAC phase, 116 patients (14.1%) had SD post-NAC, and one patient had progressive disease (PD) after receiving three cycles of docetaxel plus cisplatin (TP) NAC. Survival analysis demonstrated that patients with radiological PR had significantly worse survival compared to those with CR (HR_DFS_ = 2.40, 95% CI = 1.13–5.11, *P* = 0.019, Additional file 1: Fig. S2C), and patients with SD/PD demonstrated the worst survival outcome. Based on this finding, we classified radiological responses into 3 subgroups: CR, PR, and SD/PD. Notably, patients with tumor stage I-II (T1-2) and tumor stage III-IV (T3-4) did not show significant differences in CR and PR rates (T1-2: 8 [7.7%] CR vs. 96 [92.3%] PR; T3-4: 48 [8.0%] CR vs. 552 [92.0%] PR; *P* = 0.91). The possible explanation for the comparable distribution of CR/PR in T1-2 versus T3-4 was that only locally advanced NPC (LA-NPC) (stage III-IV) patients were included in this study, thus patients with T1-2 would have more advanced N stages.

Next, we explored the relationships between biological and radiological responses and identified that they were positively correlated, with ~95% CR patients and ~75% PR patients having their cfEBV DNA dropped to zero after NAC, respectively (*P* < 0.01; Fig. 2F, and Additional file 1: Fig. S2D). Intriguingly, we observed an inconsistency between the biological and radiological responses in a subset of patients: of 56 and 648 patients with radiological CR and PR (radiological response) after NAC, 3 (5.3%) and 160 (24.7%) patients had detectable post-NAC cfEBV DNA (non-cBR), respectively (Fig. 2F). Moreover, across 117 patients with SD/PD (radiological responses), about 45 patients (38.5%) achieved cBR after 2–4 cycles of chemotherapy (Fig. 2F). These results prompted us to hypothesize that therapeutic responses evaluated by MRI and ctDNA may reflect distinct aspects of tumor biology and sensitivity to systemic treatment.

### Biological responses are associated with post-CRT ctDNA clearance and patient long-term survival

To further understand the clinical implications of cfEBV DNA-based biological responses. We first examined the correlations between post-NAC cfEBV DNA and post-CRT ctDNA. A total of 690 (84.0%) patients with matched post-NAC and post-CRT cfEBV DNA tests were included in the analysis. Among these, 51 patients had detectable post-CRT cfEBV DNA (median, 0.81 × 10^3^ copies/mL; IQR, 0.33–4.79 × 10^3^ copies/mL). The results demonstrated that detectable post-NAC DNA had 83.6% prediction sensitivity for detectable post-CRT ctDNA (95% CI = 78.0–88.1%). The probabilities of detectable post-CRT cfEBV DNA were 14 of 464 (3.0%) and 37 of 226 (16.4%), respectively, for patients with and without cBR after NAC (*P* < 0.01, *χ*^2^ test; Additional file 1: Fig. S3A), suggesting that early cfEBV DNA kinetics was an informative indicator of whole-course treatment responses.

Next, we sought to determine the predictive value of post-NAC cfEBV DNA in long-term prognosis. Survival analysis revealed that cBR post-NAC was strongly predictive of long-term prognosis (HR_DFS_ = 3.28; 95% CI = 2.55–4.23; *P* < 0.01; Fig. 2G and Additional file 1: Fig. S3B) and was independent of other clinically relevant prognostic factors in the IPW-adjusted survival analysis (Table 3). Interestingly, we identified that post-NAC cfEBV DNA was most prominently associated with distant metastasis after adjusting for clinically significant covariates (HR_cfEBV DNA_ = 3.45 vs. HR_MRI_ = 1.71, *P*_both_ < 0.05; Table 3). In contrast, although post-NAC cfEBV DNA was also an independent predictor for locoregional recurrence, radiological response exhibited higher HR_LRFS_ compared to post-NAC cfEBV DNA, suggesting that radiological response was a more preferential predictor for locoregional recurrence (HR_cfEBV DNA_ = 1.89 vs. HR_MRI(PR vs. CR)_ = 2.70 & HR_MRI(SD/PD vs. CR)_ = 5.57; Table 3). This observation echoed with the above presumption that MRI and ctDNA reflected distinct aspects of tumor biology and sensitivity to systemic treatment.

**Table 3:** Inverse probability weighting-adjusted Cox regression of biological and radiological responses to induction chemotherapy in 821 patients with locally advanced nasopharynx of head and neck cancer

Covariates	Subgroup	DFS			OS			DMFS			LRFS		
		HR	95%CI	***P***	HR	95%CI	***P***	HR	95%CI	***P***	HR	95%CI	***P***
**Post-NAC cfEBV DNA**^a^	cBR vs. Non-cBR	2.81	2.16–3.66	**<0.01**	2.34	1.68–3.26	**<0.01**	3.45	2.43–4.91	**<0.01**	1.89	1.27–2.82	**<0.01**
**Post-NAC MRI**^a^	CR vs. PR	2.34	1.05–5.25	**0.04**	2.21	0.74–6.62	0.16	1.79	0.68–4.70	0.24	2.70	0.86–8.43	0.09
	CR vs. SD/PD	4.98	2.15–11.55	**<0.01**	5.24	1.69–16.31	**<0.01**	3.30	1.20–9.13	**0.02**	5.57	1.74–17.85	**<0.01**
**Response Phenotypes**^a^	G1: cBR+CR (References)	1.00	–	–	1.00	–	–	1.00	–	–	1.00	–	–
	G3: cBR+PR	1.52	0.69–3.33	0.30	1.52	0.54–4.34	0.43	1.03	0.40–2.63	0.95	1.93	0.61–6.09	0.26
	G4: non-cBR+PR	4.44	2.01–9.84	**<0.01**	3.56	1.23–10.29	**0.02**	3.91	1.52–10.02	**<0.01**	4.02	1.25–12.95	**0.02**
	G5: cBR+SD/PD	2.81	1.14–6.95	**0.03**	2.51	0.77–8.24	0.13	0.80	0.21–3.12	0.75	5.03	1.40–18.01	**0.01**
	G6: non-cBR+SD/PD	6.60	2.85–15.29	**<0.01**	7.18	2.391.61	**<0.01**	5.09	1.90–13.63	**<0.01**	5.04	1.48–17.11	**0.01**

*Abbreviations*: *cBR* Complete biological response, *cfEBV DNA* Cell-free Epstein-Barr virus DNA, *CI* Confidence interval, *CR* Complete response, *DFS* Disease-free survival, *DMFS* Distant metastasis-free survival, *HR* Hazard ratio, *LRFS* Locoregional relapse-free survival, *MRI* Magnetic resonance imaging, *NAC* Neoadjuvant chemotherapy, *non-cBR* Non-complete biological response, *OS* Overall survival, *PD* Progression disease, *PR* Partial responses, *SD* Stable disease

^a^The following variables were adjusted via IPW algorithm: age (<45 vs. ≥45 years), sex (male vs. female), smoking (No vs. Yes), alcohol (No vs. Yes), pretreatment EBV DNA (<2 vs. ≥2 × 10^3^ copies/mL), T stage (T1-2 vs. T3-4), N stage (N0-1 vs. N2-3), IC regimens (TPF vs. GP vs. TP vs. PF vs. others), IC cycles (2 cycles vs. 3 cycles vs. 4 cycles), CCD (<160 vs. ≥160 mg/m^2^). Two-sided *P*-values were calculated using the chi-square test

Furthermore, we found that patients with non-cBR post-NAC that finally achieved cBR at the end of the CRT still sustained worse prognoses compared to those with cBR post-NAC (HR_DFS_ = 2.70; 95% CI = 2.00–3.64; *P* < 0.01; Fig. 2H), suggesting that early biological responses were informative and that delayed ctDNA response conferred unfavorable outcomes. Moreover, among 242 patients with disease progression events, detectable cfEBV DNA post-NAC encompassed over half (122/242) of all failures, while detectable post-CRT ctDNA encompassed only 18% (39/211) of all failures (*P* < 0.05). Together, these data indicated that unfavorable biological cfEBV DNA responses at early treatment course identified an at-risk subgroup that encompassed large proportions of long-term failures.

### Biological responses provide additional prognostic information to RECIST

**Fig. 3:**
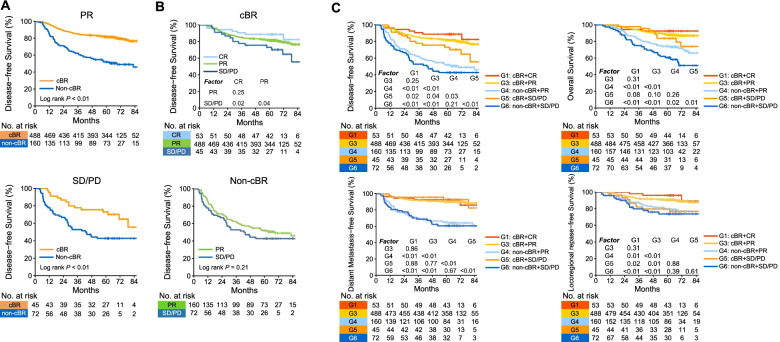
Biological responses provide additional prognostic information to RECIST. **A** Top panel: Kaplan-Meier survival plot of DFS in patients achieving RECIST PR stratified by biological responses to NAC. Bottom panel: Kaplan-Meier survival plot of DFS in patients with RECIST PD/SD stratified by biological responses to NAC. **B** Top panel: Kaplan-Meier survival plot of DFS in patients achieving cBR stratified by RECIST (CR vs. PR vs. SD/PD). Bottom panel: Kaplan-Meier survival plot of DFS in patients who did not achieve cBR stratified by RECIST (PR vs. SD/PD). **C** Kaplan-Meier survival plot of DFS, OS, DMFS, and LRFS across response phenotypes based on biological plus radiological responses to NAC. G1: cBR+CR, G2: non-cBR+CR, G3: cBR+PR, G4: non-cBR+PR; G5: cBR+SD/PD, and G6: non-cBR+SD/PD. Abbreviations: cBR, complete biological response; cfEBV DNA, cell-free Epstein-Barr virus DNA; CR, complete response; DFS, disease-free survival; DMFS, distant metastasis-free survival; HR, hazard ratio; LRFS, locoregional relapse-free survival; non-cBR, non-complete biological response; OS, overall survival; PD, progression disease; PR, partial responses; SD, stable disease

Given the above observations, we asked whether early ctDNA kinetics provided additional clinical utility beyond imaging response assessments. To answer this question, we first stratified patients according to their radiological response and investigated whether patients with RECIST CR or PR had an unfavorable prognosis when they had detectable post-NAC ctDNA. Interestingly, we identified that post-NAC cfEBV DNA further stratified PR subgroup, with non-cBR patients having significantly worse DFS (HR_DFS_ = 3.17, 95% CI = 2.36–4.25, *P* < 0.01; Fig. 3A). Unfortunately, the survival outcomes for CR subgroup (non-cBR vs. cBR) were not depicted due to the limited sample size in CR+non-cBR subgroup (*n* = 3). In addition, across patients with RECIST SD/PD, patients who achieved cBR post-NAC had more favorable DFS compared with those who did not (HR_DFS_ = 2.32; 95% CI = 1.28–4.20; *P* < 0.01; Fig. 3A).

Next, we determined whether radiological responses can further stratify patients with or without cBR. We found that radiological response further stratified patients with cBR and that patients with SD/PD had significantly worse DFS compared to those with CR (HR_DFS_ = 4.93; 95% CI = 2.25–10.82; *P* = 0.02; Fig. 3B) and PR (HR_DFS_ = 2.06; 95% CI = 1.52–2.78; *P* = 0.04), whereas the difference was not significant between CR versus PR (*P* > 0.05), possibly attributed to the limited events, given that cBR patients had superior prognosis compared to the overall cohort (Additional file 1: Fig. S2C). In addition, across the non-cBR subgroups, although patients with PR demonstrated better prognosis compared to those with SD/PD, the differences did not reach statistical significance for DFS (*P* > 0.05; Fig. 3B), suggesting that patients who did not successfully achieve biological response (non-cBR) would have equally inferior long-term tumor control regardless of radiological PR or SD/PD.

Based on the above observation, we further combined the radiological and biological response subgroups and yielded 6 response phenotypes: G1 (cBR+CR, *n* = 53, 6.5%), G2 (non-cBR+CR, *n* = 3, 0.4%), G3 (cBR+PR, *n* = 488, 59.4%), G4 (non-cBR+PR, *n* = 160, 19.5%); G5 (cBR+SD/PD, *n* = 45, 5.5%), and G6 (non-cBR+SD/PD, *n* = 72, 8.8%). Across diverse phenotypes, we next mainly focused our following analysis on phenotypes with contradictory biological and radiological response evaluations (G4 [non-cBR+PR] and G5 [cBR+SD/PD]). For G4, one important issue here was whether non-cBR was potentially confounded by false-positive cfEBV DNA tests. To address this point, we further compared their baseline characteristics with G3 (cBR+PR) and identified that patients with non-cBR+PR response phenotype tended to have higher clinical stages and baseline cfEBV DNA load (Additional file 2: Table S2). Interestingly, even adjusting for clinical covariates in multivariate analysis, patients with non-cBR+PR still had significantly worse prognosis in all endpoints compared to cBR+PR (Table 4), suggesting that detectable cfEBV DNA for patients with PR was clinically informative, rather than just confounded by false-positive tests. Analogously, to further address whether cBR was potentially confounded by false-negative cfEBV DNA tests for patients with SD/PD in G5, we further compared their baseline characteristics with G6 (non-cBR+SD/PD) and observed that they had lower pretreatment cfEBV DNA compared to G6 (Additional file 2: Table S3). Interestingly, after adjusting for clinically relevant covariates, patients with cBR+SD/PD (G5) still harbored significantly better prognosis in OS, DFS, and DMFS compared to non-cBR+SD/PD (G6) (Table 5). Notably, the differences in DMFS were most prominent (HR_DMFS_ = 5.81, 95% CI = 2.09–16.18, *P* < 0.01), whereas the difference in LRFS did not reach statistical significance (*P* > 0.05). These data indicated that undetectable cfEBV DNA for patients with SD/PD was clinically informative rather than just confounded by false-negative tests, especially in forecasting better distant control across patients with SD/PD, but not for local control. Collectively, we revealed that the contradictory biological and radiological responses bred additional valuable prognostic information.

**Table 4:** Inverse probability weighting-adjusted Cox regression between cBR+PR and non-cBR+PR subgroups in locally advanced nasopharynx of head and neck cancer

Covariates	Subgroup	DFS			OS			DMFS			LRFS		
		HR	95%CI	***P***	HR	95%CI	***P***	HR	95%CI	***P***	HR	95%CI	***P***
**Response Phenotypes**^a^	G3: cBR+PR (References)	1.00	–	–	1.00	–	–	1.00	–	–	1.00	–	–
	G4: non-cBR+PR	2.95	2.18–4.00	**<0.01**	2.43	1.65–3.58	**<0.01**	3.86	2.62–5.68	**<0.01**	1.98	1.25–3.14	**<0.01**

*Abbreviations*: *cBR* Complete biological response, *cfEBV DNA* Cell-free Epstein-Barr virus DNA, *CI* Confidence interval, *DFS* Disease-free survival, *DMFS* Distant metastasis-free survival, *HR* Hazard ratio, *LRFS* Locoregional relapse-free survival, *MRI* Magnetic resonance imaging, *NAC* Neoadjuvant chemotherapy, *non-cBR* Non-complete biological response, *OS* Overall survival, *PD* Progression disease, *PR* Partial responses

^a^The following variables were adjusted via IPW algorithm: age (<45 vs. ≥45 years), sex (male vs. female), smoking (No vs. Yes), alcohol (No vs. Yes), pretreatment EBV DNA (<2 vs. ≥2 × 10^3^ copies/mL), T stage (T1-2 vs. T3-4), N stage (N0-1 vs. N2-3), IC regimens (TPF vs. GP vs. TP vs. PF vs. others), IC cycles (2 cycles vs. 3 cycles vs. 4 cycles), CCD (<160 vs. ≥160 mg/m^2^). Two-sided *P*-values were calculated using the chi-square test

**Table 5:** Inverse probability weighting-adjusted Cox regression between cBR+SD/PD and non-cBR+SD/PD subgroups in locally advanced nasopharynx of head and neck cancer

Covariates	Subgroup	DFS			OS			DMFS			LRFS		
		HR	95%CI	***P***	HR	95%CI	***P***	HR	95%CI	***P***	HR	95%CI	***P***
**Response Phenotypes**^a^	G5: cBR+SD/PD (References)	1.00	–	–	1.00	–	–	1.00	–	–	1.00	–	–
	G6: non-cBR+SD/PD	2.48	1.37-4.50	**<0.01**	2.59	1.29-5.20	**<0.01**	5.81	2.09-16.18	**<0.01**	1.32	0.57-3.07	0.52

*Abbreviations*: *cBR* Complete biological response, *cfEBV DNA* Cell-free Epstein-Barr virus DNA, *CI* Confidence interval, *DFS* Disease-free survival, *DMFS* Distant metastasis-free survival, *HR* Hazard ratio, *LRFS* Locoregional relapse-free survival, *MRI* Magnetic resonance imaging, *NAC* Neoadjuvant chemotherapy, *non-cBR* Non-complete biological response, *OS* Overall survival, *PD* Progression disease, *SD* Stable disease

^a^The following variables were adjusted via IPW algorithm: age (<45 vs. ≥45 years), sex (male vs. female), smoking (No vs. Yes), alcohol (No vs. Yes), pretreatment EBV DNA (<2 vs. ≥2 × 10^3^ copies/mL), T stage (T1-2 vs. T3-4), N stage (N0-1 vs. N2-3), IC regimens (TPF vs. GP vs. TP vs. PF vs. others), IC cycles (2 cycles vs. 3 cycles vs. 4 cycles), CCD (<160 vs. ≥160 mg/m^2^). Two-sided *P*-values were calculated using the chi-square test

Finally, we asked whether patients with biological cBR plus radiological SD/PD (G5) would have comparable survival with patients who achieved radiological PR plus biological non-cBR (G4). To our surprise, G5 had significantly more favorable long-term prognosis compared to G4, especially in the control of distant metastasis (*P*_all_ < 0.05; Fig. 3C).

### Combinations of biological and radiological responses refine risk groupings

**Fig. 4:**
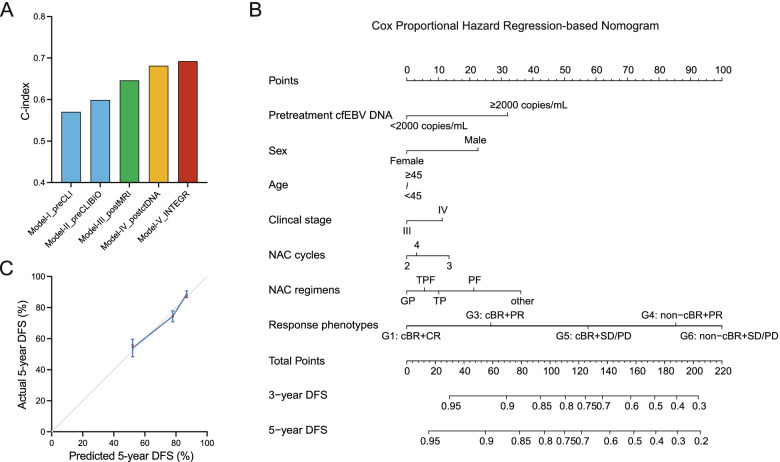
The combinations of biological and radiological responses refine risk groupings. **A** Bar plot showing the C-index and 95% CI for predicting the 5-year DFS by five models incorporating pretreatment risk factors with/without ctDNA and on-treatment parameters using the CpH method. **B** Nomogram for predicting the 3- and 5-year DFS, which integrated conventional pretreatment risk factors with pretreatment ctDNA, radiological and ctDNA-based response phenotypes, and therapeutic information. The total point values were independently calculated and then applied to the corresponding probability scale. **C** Calibration plots showing the actual risk probability by decile (*y*-axis) over the nomogram-predicted risk probability (*x*-axis). Abbreviations: cBR, complete biological response; cfEBV DNA, cell-free Epstein-Barr virus DNA; CR, complete response; DFS, disease-free survival; non-cBR, non-complete biological response; PD, progression disease; PR, partial responses; SD, stable disease

Given the above findings that cfEBV DNA harbored critical biological information and that its on-treatment clearance kinetics identified preferentially at-risk populations beyond the traditional imaging evaluations, we presumed that inclusion of ctDNA testing would refine the risk estimates across patients with similar initial risks based on clinically relevant factors; moreover, as therapy is introduced, further risk stratification considering the on-treatment ctDNA measurement, radiological response, and therapeutic information would refine personalized dynamic risk estimates. To test this hypothesis, we established five risk prediction models incorporating clinically important factors with/without ctDNA and on-treatment parameters (Fig. 4A). The models were constructed based on Cox proportional hazard regression (CpH) model.

In the first model, three parameters (sex, age, clinical stage) established from prior literature or datasets were initially incorporated (Model-I: pretreatment clinical [Model-I_preCLI]). We determined the performance of the model for predicting 5-year DFS, a clinically relevant milestone and standard endpoint in cancer, and identified a bias-corrected Harrell’s concordance index (C-index) of 0.57. Importantly, the predictive accuracy of 5-year DFS significantly improved when pretreatment cfEBV DNA was incorporated (Model-II: pretreatment clinic-biological [Model-II_preCLIBIO]), with the C-index reaching 0.60. Next, we introduced treatment information and radiological/biological response parameters into the model (Model III-V). Model-III_postMRI, incorporating treatment information and radiological responses, had a significantly improved C-index of 0.65, and the C-index of Model IV (Model-IV_postctDNA), which incorporated treatment information and biological responses, was 0.68. Finally, given the above observation that on-treatment MRI and ctDNA reflected distinct aspects of tumor biology and sensitivity to systemic treatment, we established Model-V (Model-V_INTEGR), which integrated pretreatment factors with radiological and ctDNA-based response phenotypes, and therapeutic information. The C-index of Model-V reached 0.69.

Given that Model-V_INTEGR outperformed the models using pretreatment risk factors or on-treatment radiological assessments, we further developed a nomogram for quantifying the 3- and 5-year risks of disease progression in patients with diverse pretreatment and on-treatment features (Fig. 4B). The calibration plots indicated good agreement between the model’s predicted and observed survival estimates (Fig. 4C).

## Discussion

Tumor responses following systemic treatment demonstrate significant intertumoral heterogeneity over time, which informs the dynamic risk probabilities for individual patients. The conventional approach for capturing phenotypic responses has been RECIST-based imaging examinations, which are inherently limited by the difficulty of on-schedule collection, expensive cost, and stratification inaccuracy. Our analyses of the performance of on-treatment cfEBV DNA to systemic treatment in a large cohort of patients with NPC sheds light on this issue by demonstrating the feasibility and enhanced prognostication of ctDNA-based biological response evaluation and risk stratification, which provides critical information not only complementary to but also beyond RECIST. Our findings add to the body of prior knowledge that patient risk probabilities are dynamic and can be refined with the introduction of biological response information, which will facilitate future risk-adapted clinical trial designs for personalized therapeutic approaches.

Previous risk stratification of patients with cancer has primarily focused on pretreatment factors, and therapeutic decisions are made before treatment initiation and remain unchanged throughout the whole treatment course [21, 22]. Nevertheless, in the present research, we demonstrated and proposed that early on-treatment tumor responses, not only radiological response but also ctDNA-based biological responses, harbored critical prognostic information and that incorporating these factors greatly enhanced patient risk stratification. The refined and dynamic on-treatment risk stratification strategy would consequently open the door to risk-adapted treatment intensification/de-intensification for personalized medicine. Taking the NPC model as an example, stage III–IV_A_ NPC are classified as locally advanced NPC with high risks, and standard treatment for LA-NPC entails NAC plus CRT [23, 24]. Although a large body of phase III clinical trials supports its clinical efficacy [25–27], in clinical settings, it cannot be neglected that a subset of patients who responded well to NAC had decreased recurrence risk and would be overtreated during the subsequent intensive CRT. In contrast, another proportion of patients who harbored chemotherapy-resistant tumor clones did not respond well to NAC, and experienced treatment failure soon after the completion of CRT. These suggest that the current pretreatment-determined, one-size-fits-all treatment strategy is suboptimal. Based on our on-treatment cfEBV DNA-based risk model, we would carefully propose a risk-adapted clinical approach. Specifically speaking, when patients with predicted 5-year overall survival rates over 90% based on the on-treatment risk model, it would be intuitive to de-intensify treatment to avoid unnecessary adverse events and improve quality of life, given that the accumulating evidences support the premise that LA-NPC with relatively low risk may be less beneficial to intensive concurrent chemotherapy [28], and two cycles of concurrent cisplatin share equal survival outcomes but significantly reduced adverse events with three cycles [29]. Moreover, for at-risk patients with predicted 5-year overall survival rates less than 90% after NAC, it is promising to intensify treatment in appropriate ways (i.e., additional adjuvant chemotherapy). Especially, for those with predicted 5-year overall survival rates lower than that of pretreatment, which may suggest insensitivity to prior chemotherapy, it is of urgent need to alter chemotherapeutic regimens during the following therapeutic phases and/or integrate novel treatment modalities (i.e., immune checkpoint inhibitors and/or target therapy) in order to circumvent chemoresistant clones and improve survival [30, 31]. Notably, the clinical feasibility of the above interventions ought to be carefully tested in prospective clinical trials.

There have been increasing efforts to combine on-treatment response-based parameters with pretreatment factors for refined risk stratification. In routine clinical practice, physicians often utilize serial anatomical imaging or biopsy information for response assessments [32, 33]. Unfortunately, these parameters do not easily lend themselves to longitudinal examinations due to the difficulty of on-schedule collection, expensive cost, and invasiveness. Moreover, we determined that RECIST-based imaging evaluation did not fully capture patients with favorable prognosis and clinical benefit, which is in line with recently published research [9, 10]. To overcome this barrier, we demonstrated the feasibility and enhanced prognostication of minimally invasive ctDNA-based biological response evaluation in a large cohort of patients with cancer being treated consistently. Furthermore, we found 25.3% discordance between the ctDNA and imaging-based response evaluations and that early ctDNA kinetics provided critical information not only complementary to but also beyond RECIST for identifying patients with long-term tumor control, especially those with SD/PD but cBR, who would still derive meaningful clinical benefit from treatment. This finding is clinically informative, as current strategies for identifying a responsive population with clinical and radiological methods at early timepoints are suboptimal. Notably, in the context of immunotherapy, patients who met the criteria for PD based on RECIST were noted to have late but deep and durable responses [34]. Further studies are warranted to examine whether serial ctDNA surveillance may facilitate the timely recognition of patients who would benefit from ICIs or other treatment modalities.

In recent years, ctDNA has revolutionized the management of patients with cancer due to its minimally invasive nature and access to cancer-specific information. Nevertheless, previous studies have largely focused on the implications of pretreatment ctDNA. Although low baseline ctDNA levels are widely reported to be associated with favorable clinical outcomes, we and others have found that the effect size is generally modest compared to the on-treatment counterpart [12, 35]. Moreover, we identified that post-NAC cfEBV DNA levels, but not baseline cfEBV DNA levels strongly correlated with RECIST, suggesting that other mechanisms underpinning clonal sensitivity to treatment are likely implicated in the on-treatment, but not baseline, ctDNA. Therefore, it is not surprising that the model incorporating on-treatment cfEBV DNA changes and pretreatment cfEBV DNA factors demonstrated the highest prognostication effect compared to the model incorporating pretreatment cfEBV DNA alone. On this note, we propose that on-treatment cfEBV DNA kinetics provide critical information not only complementary to pretreatment cfEBV DNA, but also beyond it, which ought to be included in future prognostic/predictive models. Here, it is also worth noting that although we adopted cfEBV DNA in NPC as a model in this research to unveil the application of on-treatment ctDNA, we cannot exclude the possibility that there are nasopharyngeal carcinoma cells that do not have EBV integrations and that except for EBV DNA, other types of cancer-associated DNA (i.e., point mutations, copy number aberrations, alterations in DNA methylation) can also be released into the plasma of NPC patients. Especially, for NPC patients that are not associated with EBV infections, detections of genetic and epigenetic markers other than cfEBV DNA for risk monitoring are challenging and merit future investigations.

Limitations of this study included lack of sequencing data in the cfEBV DNA assay design. However, this is beyond the scope of our study. According to previously published research, such information is important when distinguishing healthy controls from patients with NPC, given that the EBV DNA fragments of patients have longer fragment lengths and higher methylation levels compared to that of people without NPC [36, 37]. Second, the radiological response evaluation in NPC using RECIST criteria is rather tricky compared to other solid tumors. The skull base was not included as target lesions here, given their inferiority in reproducible repeated measurements, and the well-documented challenges of MRI in evaluating the shrinkage of skull base lesions [38]. Third, the number of patients with non-cBR+CR was limited, which made it difficult to perform survival analysis. Hence, further investigation on this subgroup is warranted to determine whether it is attributed to the false-positive cfEBV DNA test or is truly clinically informative in this subgroup. Fourth, the prognostic model in the present study was not externally validated. Therefore, the applicability of this model should be interpreted with caution; external validation cohorts and prospective studies are warranted to further confirm the clinical applicability of our model. Except for the above limitations, we revealed the values of on-treatment cfEBV DNA kinetics and their clinical utility for response evaluation and dynamic risk stratification in a cohort with homogeneous treatment modality and mature follow-up data. An additional strength is that we adopted the IPW algorithm to examine and compare the prognostic values of cfEBV DNA and other variables, which takes the advantage of efficiently simulating a randomized trial in time-to-event analyses without sacrificing sample size and statistical power, which enhanced the validity of our findings.

## Conclusions

In summary, we reveal the clinical utility of ctDNA-based testing in therapeutic response evaluation for a refined risk stratification, which informs early clinical benefits and adds value not only complementary to but also beyond the RECIST-based imaging response evaluation. The dynamic and refined risk profiling can inform future risk-adapted personalized therapy.

## Methods

### Patient population and study design

The medical records of 10,126 non-metastatic nasopharynx of head and neck cancer patients who had been diagnosed between April 2009 and December 2015 and received radical intent treatment were screened from a cancer-specific database within the big-data intelligence framework at Sun Yat-sen University Cancer Center (SYSUCC). We have provided a detailed description of this big-data platform previously [39]. We identified a total of 821 patients with LA-NPC who consistently received standard therapy (NAC plus concurrent CRT), and had on-treatment biological (plasma cfEBV DNA) and radiological (magnetic resonance imaging [MRI]) assessments upon completing NAC. The inclusion criteria were as follows: (1) World Health Organization (WHO) type II or III NPC, positive for EBV viral capsid antigen (VCA/IgA) or EBV early antigen (IgA/EA); (2) received standard NAC plus CRT; (3) had detectable pretreatment EBV DNA (>0 copies/mL); (4) had biomarker surveillance of cfEBV DNA quantification upon completing 2–4 cycles of NAC; and (5) had MRI assessment upon completing NAC. In this research, to account for treatment heterogeneity and reduce potential bias, we restricted the NAC regimens to docetaxel-cisplatin-fluorouracil (TPF), TP, cisplatin-fluorouracil (PF), and gemcitabine-cisplatin (GP), and the concurrent chemotherapy regimens to cisplatin and nedaplatin, according to the published evidence and clinical trials [40–42]. The collection schema and stepwise selection process are presented in Fig. 1.

### Diagnosis and staging

All patients underwent pretreatment evaluations, including physical examinations, hematology and biochemistry examinations, plasma cfEBV DNA testing, fiberoptic nasopharyngoscopy, MRI of the nasopharynx and neck, computed tomography (CT) of the chest and abdomen, whole-body bone scan, or 18F-fluorodeoxyglucose positron emission tomography–CT (PET-CT). Patients were staged according to the 8th edition of the American Joint Commission on Cancer (AJCC) staging system by two experienced radiation oncologists specializing in head and neck cancer [43]; any disagreements were resolved through internal discussion.

### Treatment protocol

The standard treatment for advanced-stage NPC is NAC plus CRT according to the recommendations of the updated National Comprehensive Cancer Network (NCCN) guideline [44], and all patients included in the present study consistently received NAC plus CRT. We have described the treatment protocols and dose modifications previously [12].

### Radiological assessment of tumor response

Patient objective radiological responses to NAC were evaluated using MRI post-NAC according to RECIST 1.1 [38]. Patients were classified as CR with the following: (a) disappearance of all target lesions; (b) each target lymph node must have reductions in short axis to <1.0 cm. PR was defined as having at least 30% decrease in the sum of the longest diameter for all target lesions plus the sum of the short axis of all target lymph nodes (post-baseline sum of the dimensions, PBSD). PD was defined as follows: (a) at least one new malignant lesion, which also included any lymph node that was normal at baseline (<1.0 cm short axis) and that had increased to ≥1.0 cm short axis during evaluation; (b) at least a 20% increase in PBSD. In addition, the PBSD must also demonstrate an absolute increase of at least 0.5 cm from the minimum sum of the dimensions. Patients were classified as SD if there was neither sufficient shrinkage to qualify for PR, nor sufficient increase to qualify for PD. “Responders” referred to patients with CR and PR; “non-responders” referred to patients with SD and PD.

### Plasma cfEBV DNA surveillance and quantification

#### Real-time quantitative polymerase chain reaction (RT-qPCR)

Peripheral blood (3.0 mL) was collected from each patient at the following timepoints: within 2 weeks before treatment initiation; 17~21 days after NAC completion and before the initiation of CRT, within 3 months after CRT completion. Plasma cfEBV DNA was quantified with RT-qPCR targeting the *Bam*HI W fragment of the EBV genome as described by Lo et al. [45]. The sequences of the forward and reverse primers were CCCAACACTCCACCACACC and TCTTAGGAGCTGTCCGAGGG, respectively. A dual fluorescently labelled oligomer, 5′-(FAM) CACACACTACACACACCCACCCGTCTC (TAMRA)-3′ served as the probe. Amplifications were performed in an Applied Biosystems 7700 Sequence Detector and analyzed using Sequence Detection System software (version 1.6.3) developed by Applied Biosystems. The plasma EBV DNA concentration was calculated as previously described [45]. All samples were analyzed in duplicate and were re-tested a third time if the first two tests yielded varying results. The operators who measured the ctDNA were blinded to the patient information and clinical outcomes.

#### Cut point identification

Based on our previous work, the cut-off cfEBV DNA level in treatment-naïve samples was 2000 copies/mL, as sensitivity analysis revealed stable HR for survival endpoints in the presence of increasing cfEBV DNA levels, and 2000 copies is a stable and robust cut-off in the pretreatment setting [17]. The cut-off on-treatment and post-treatment cfEBV DNA levels were scored based on a detectable/undetectable scale according to published research [46, 47].

### Post-treatment surveillance

Patients were followed-up every 3 months during the first 2 years and every 6 months for 3 years thereafter. During the clinical visit, routine physical examinations and fiberoptic nasopharyngoscopy were performed. Patients who were clinically suspected to have disease recurrence were recommended to undergo further examinations; confirmatory cytological biopsies were performed if possible.

### Statistical analysis

#### Clinical endpoints

We focused on patient biological response to NAC evaluated by plasma cfEBV DNA, the associations with the radiological response, and the clinical implications. The primary survival endpoint was DFS, defined as the time from treatment initiation to tumor progression or death. The secondary endpoint was OS, DMFS, and LRFS.

#### IPW algorithm

Survival analysis was performed using the Kaplan-Meier method and compared by the log-rank test [48]. CpH was used for estimating the HR [49]. Given that the unbalanced covariates across subgroups may potentially bias the findings, we calculated the adjusted HRs of the variables of interest for each survival endpoint, adjusting for clinically important covariates using the IPW algorithm [50, 51]. IPW is a propensity score-based algorithm that can efficiently balance the confounding factors across the subgroups of interest [52]. The rationales for choosing IPW method are that it generates balanced distributions of covariates across subgroups of “variable of interest”, by weighting each patient using a propensity score [52]. It can well-simulate the properties of a randomized clinical trial design [53, 54], and the adjusted efficacy can be easily checked by calculating the distributions of “covariates” before and after IPW adjustment across subgroups of “variable of interest” (as shown in Table 2), whereas in a traditional multivariable regression model, this data was rarely evaluated. In addition, the inversed propensity score embedded in the IPW method takes advantages over conventional propensity score-based algorithm, in that it keeps all patients in the analysis. Therefore, it takes the advantage of unbiased estimations without sacrificing sample size and statistical power [55, 56]. The robustness of the IPW approach was validated by comparing the baseline characteristics across the IPW-adjusted subgroups.

#### Model development

To investigate the concept that inclusion of ctDNA testing and on-treatment measurement of tumor responses would refine the dynamic risk estimation across patients with similar initial risks, we developed and compared the predictive ability of five prognostic models, incorporating well-established prognostic factors with or without ctDNA and/or therapeutic information. With the aim of evaluating the clinical applicability of the findings and increasing prediction accuracy, we adopted CpH to predict individualized survival. The proportional hazards assumption was verified based on the Schoenfeld residuals [57]. We assessed the performance of the CpH model using Harrell’s C-index and visualized with calibration plots as previously described [17]. The performance of the models was compared based on their discrimination and calibration.

#### Statistical considerations

The baseline characteristics among the groups were compared using the *χ*^2^ test (Fisher’s exact test or Pearson’s *χ*^2^ test where appropriate) for categorical variables, and with the Mann-Whitney *U* test or Kruskal-Wallis test for continuous variables. All statistical tests were two-sided; *P* < 0.05 was considered statistically significant. The analyses were performed in R (version 3.4.4; http://www.r-project.org/) and SPSS (version 23.0; SPSS Inc.).

## Supplementary Information

**Additional file 1: Fig. S1.** The course of patients with NPC through treatment and surveillance. **Fig. S2.** Biological response to NAC and the correlations with radiological response. **Fig. S3.** Biological responses are associated with post-CRT ctDNA clearance and long-term survival.

**Additional file 2: Table S1.** Prognostic values of pretreatment circulating tumor DNA in 821patients with locally advanced nasopharynx of head and neck cancer. **Table S2.** Comparison of baseline characteristics between cBR+PR and non-cBR+PR subgroups. **Table S3.** Comparison of baseline characteristics between cBR+SD/PD and non-cBR+SD/PD subgroups.

## Data Availability

The data have been deposited in the Research Data Deposit public platform (accession code: RDDA2021002022). Data supporting the findings of this study are available from the corresponding author upon reasonable request.

## Abbreviations

AJCC: American Joint Commission on Cancer
cBR: Complete biological response
cfEBV DNA: Cell-free Epstein-Barr virus DNA
CpH: Cox proportional hazard regression
CR: Complete response
CRT: Chemoradiotherapy
CT: Computed tomography
DFS: Disease-free survival
DMFS: Distant metastasis-free survival
GP: Gemcitabine-cisplatin
IPW: Inverse probability weighting
LA-NPC: Locally advanced nasopharyngeal carcinoma
LRFS: Locoregional relapse-free survival
NAC: Neoadjuvant chemotherapy
NCCN: National Comprehensive Cancer Network
Non-cBR: Non-complete biological response
NPC: Nasopharyngeal carcinoma
OS: Overall survival
PD: Progression disease
PET-CT: 18F-fluorodeoxyglucose positron emission tomography–CT
PF: Cisplatin-fluorouracil
PSM: Propensity score matching
RECIST: Response Evaluation Criteria in Solid Tumors
SD: Stable disease
TP: Docetaxel-cisplatin
TPF: Docetaxel-cisplatin-fluorouracil
