# Supplementary material for: Improving on-treatment risk stratification of cancer patients with refined response classification and integration of circulating tumor DNA kinetics
Source: BMC Med. 2022 Aug 23;20:268. doi: 10.1186/s12916-022-02463-5 (PMC9396864; doi:10.1186/s12916-022-02463-5)
Supplement: Supplementary file 1 — Additional file 1: Fig. S1. The course of patients with NPC through treatment and surveillance. Fig. S2. Biological response to NAC and the correlations with radiological response. Fig. S3. Biological responses are associated with post-CRT ctDNA clearance and long-term survival. [file 12916_2022_2463_MOESM1_ESM.docx]

**Additional File 1:**

**Improving Ontreatment Risk Stratification of Cancer Patients with Refined Response Classification and Integration of Circulating Tumor DNA Kinetics**


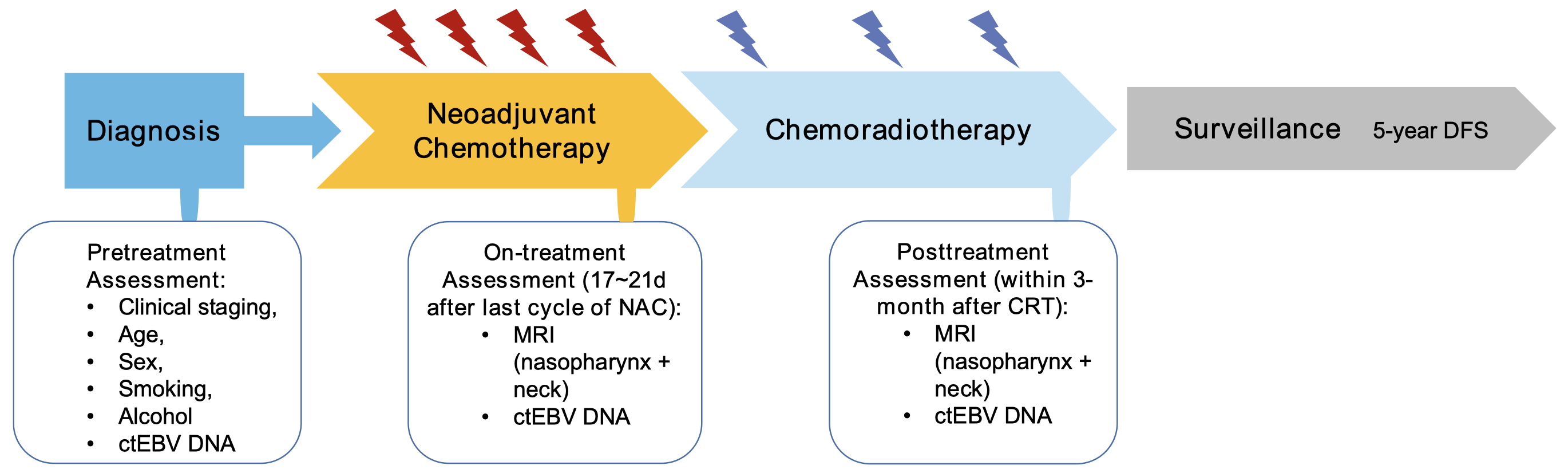


**Fig. S1. The course of patients with NPC through treatment and surveillance.**

Pretreatment risk factors were obtained routinely prior to treatment initiation. Interim response information was assessed with MRI and cfEBV DNA after NAC completion. Patients were followed-up for over 5 years after treatment completion.

Abbreviations: CRT, chemoradiotherapy; ctEBV DNA, circulating tumor Epstein-Barr virus DNA; DFS, disease-free survival; DMFS, distant metastasis-free survival; HR, hazard ratio; NAC, neoadjuvant chemotherapy.


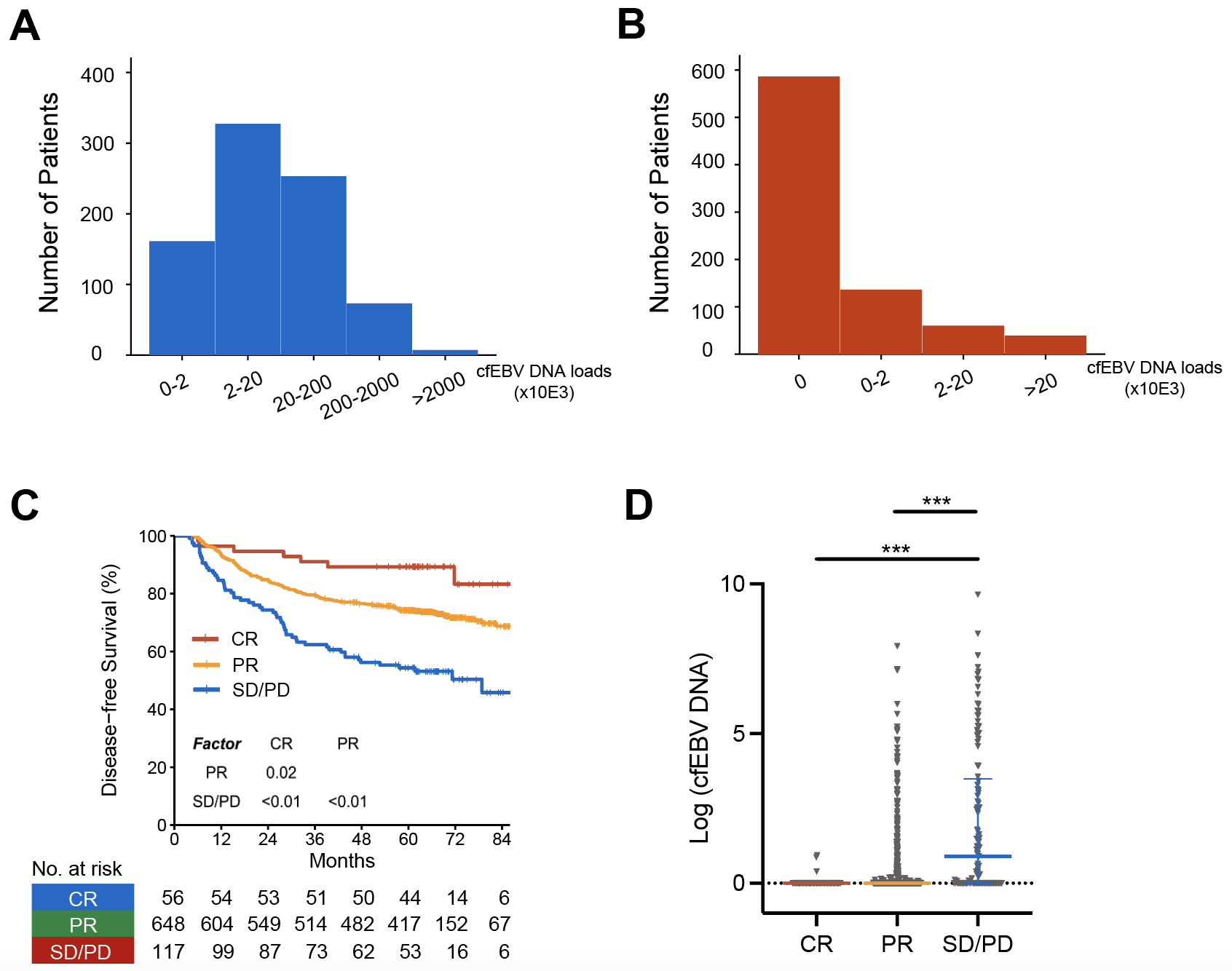


**Fig. S2. Biological response to NAC and the correlations with radiological response.**

A, Distribution of pretreatment cfEBV DNA in 821 patients with LA-NPC.

B, Distribution of post-NAC cfEBV DNA in 821 patients with LA-NPC.

C, Kaplan-Meier survival plot of DFS in patients with different radiological responses (CR versus. PR versus. SD/PD).

D, Distribution of post-NAC cfEBV DNA levels across patients with RECIST CR, PR, SD/PD.

Abbreviations: cfEBV DNA, cell-free Epstein-Barr virus DNA; CR, complete response; DFS, disease-free survival; LA-NPC, locally advanced nasopharyngeal carcinoma; NAC, neoadjuvant chemotherapy; PD, progression disease; PR, partial responses; SD, stable disease.


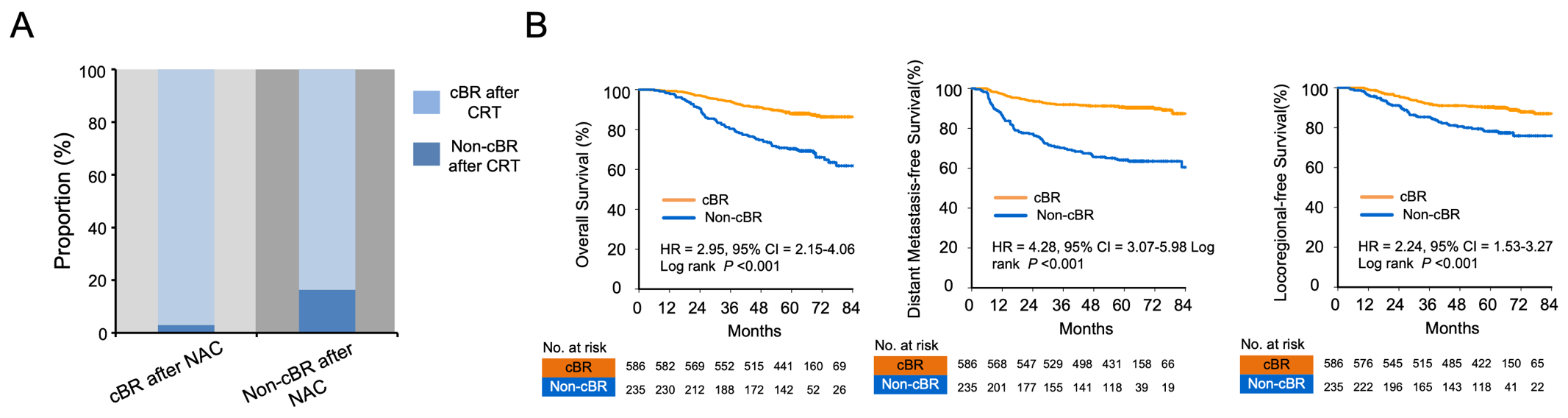


**Fig. S3. Biological responses are associated with post-CRT ctDNA clearance and long-term survival.**

A, The associations of post-NAC cfEBV DNA responses with cfEBV DNA clearance at the end of CRT.

B, Kaplan-Meier survival plot of OS, DMFS, and LRFS in patients with cBR versus non-cBR post-NAC.

Abbreviations: cBR, complete biological response; CI, confidence interval; CRT, chemoradiotherapy; ctDNA, circulating tumor DNA, DFS, disease-free survival; DMFS, distant metastasis-free survival; HR, hazard ratio; NAC, neoadjuvant chemotherapy; non-cBR, non-complete biological response.
