# Supplementary material for: Improving on-treatment risk stratification of cancer patients with refined response classification and integration of circulating tumor DNA kinetics
Source: BMC Med. 2022 Aug 23;20:268. doi: 10.1186/s12916-022-02463-5 (PMC9396864; doi:10.1186/s12916-022-02463-5)
Supplement: Supplementary file 2 — Additional file 2: Table S1. Prognostic values of pretreatment circulating tumor DNA in 821patients with locally advanced nasopharynx of head and neck cancer. Table S2. Comparison of baseline characteristics between cBR+PR and non-cBR+PR subgroups. Table S3. Comparison of baseline characteristics between cBR+SD/PD and non-cBR+SD/PD subgroups. [file 12916_2022_2463_MOESM2_ESM.docx]

**Additional File 2:**

**Improving Ontreatment Risk Stratification of Cancer Patients with Refined Response Classification and Integration of Circulating Tumor DNA Kinetics**

**Table S1: Prognostic values of pretreatment circulating tumor DNA in 821patients with locally advanced nasopharynx of head and neck cancer**

| **Covariates** | **Subgroup** | **DFS** | | |  | **OS** | | |  | **DMFS** | | |  | **LRFS** | | |
| --- | --- | --- | --- | --- | --- | --- | --- | --- | --- | --- | --- | --- | --- | --- | --- | --- |
|  |  | **HR** | **95%CI** | ***P*** |  | **HR** | **95%CI** | ***P*** |  | **HR** | **95%CI** | ***P*** |  | **HR** | **95%CI** | ***P*** |
| **Pre cfEBV DNA**^†^  **(copy/mL)** | Continuous | 1.08 | 1.03-1.14 | <0.01 |  | 1.07 | 1.01-1.13 | 0.03 |  | 1.13 | 1.06-1.19 | <0.01 |  | 1.03 | 0.96-1.11 | 0.38 |
| **Pre cfEBV DNA**^†^  **(copy/mL)** | <2000 vs. ≥2000 | 2.03 | 1.39-2.98 | <0.01 |  | 1.844 | 1.16-2.94 | 0.01 |  | 2.51 | 1.46-4.32 | <0.01 |  | 1.86 | 1.09-3.17 | 0.02 |

Abbreviations: cfEBV DNA, cell-free Epstein-Barr virus DNA; CI, confidence interval; DFS, disease-free survival; DMFS, distant metastasis-free survival; HR, hazard ratio; LRFS, locoregional relapse-free survival; OS, overall survival.

^†^ The following variables were adjusted in the Cox regression model: age (<45 vs. ≥45 years), sex (male vs. female), smoking (No vs. Yes), alcohol (No vs. Yes), T stage (T1-2 vs. T3-4), N stage (N0-1 vs. N2-3), IC regimens (TPF vs. GP vs. TP vs. PF vs. others), IC cycles (2 cycles vs. 3 cycles vs. 4 cycles), CCD (<160vs. ≥160 mg/m^2^).

**Table S2: Comparison of baseline characteristics between cBR+PR and non-cBR+PR subgroups**

| **Characteristics** | **cBR+PR**  **(N = 488)** | **Non-cBR+PR**  **(N = 160)** | ***P^†^*** |
| --- | --- | --- | --- |
|  | **No. (%)** | **No. (%)** |  |
| Age, years |  |  | 0.32 |
| <45 | 289 (59.2) | 87 (54.4) |  |
| ≥45 | 199 (40.8) | 73 (45.6) |  |
| Sex |  |  | 0.15 |
| Male | 354 (72.5) | 126 (78.8) |  |
| Female | 134 (27.5) | 34 (21.2) |  |
| Histology, WHO type^‡^ |  |  | 0.39 |
| II | 10 (2.0) | 1 (0.6) |  |
| III | 478 (98.0) | 159 (99.4) |  |
| Smoking |  |  | 0.20 |
| No | 316 (64.8) | 94 (58.8) |  |
| Yes | 172 (35.2) | 66 (41.2) |  |
| Alcohol |  |  | 0.86 |
| No | 413 (84.6) | 137 (85.6) |  |
| Yes | 75 (15.4) | 23 (14.4) |  |
| Clinical stage^§^ |  |  | <0.01 |
| III | 231 (47.3) | 52 (32.5) |  |
| IV | 257 (52.7) | 108 (67.5) |  |
| T stage^§^ |  |  | <0.01 |
| T1 | 31 (6.4) | 7 (4.4) |  |
| T2 | 30 (6.1) | 28 (17.5) |  |
| T3 | 250 (51.2) | 60 (37.5) |  |
| T4 | 177 (36.3) | 65 (40.6) |  |
| N stage^§^ |  |  | 0.01 |
| N0 | 23 (4.7) | 4 (2.5) |  |
| N1 | 197 (40.4) | 49 (30.6) |  |
| N2 | 157 (32.2) | 51 (31.9) |  |
| N3 | 111 (22.7) | 56 (35.0) |  |
| Pre cfEBV DNA  (copies/mL) |  |  | <0.01 |
| <2 × 10^3^ | 106 (21.7) | 19 (11.9) |  |
| ≥2 × 10^3^ | 382 (78.3) | 141 (88.1) |  |
| NAC regimens |  |  | 0.65 |
| TPF | 307 (62.9) | 97 (60.6) |  |
| TP | 108 (22.1) | 35 (21.9) |  |
| GP | 36 (7.4) | 11 (6.9) |  |
| PF | 32 (6.6) | 13 (8.1) |  |
| Others^¶^ | 5 (1.0) | 4 (2.5) |  |
| NAC cycles |  |  | 0.51 |
| 2 cycles | 220 (45.1) | 79 (49.4) |  |
| 3 cycles | 245 (50.2) | 76 (47.5) |  |
| 4 cycles | 23 (4.7) | 5 (3.1) |  |

Abbreviations: cBR, complete biological response; cfEBV DNA, cell-free Epstein-Barr virus DNA; GP, gemcitabine and cisplatin; N, node; NAC, neoadjuvant chemotherapy; non-cBR, non-complete biological response; PF, cisplatin and 5-fluorouracil; PR, partial responses; T, tumor; TP, docetaxel and cisplatin; TPF, docetaxel, cisplatin, and 5-fluorouracil; WHO, World Health Organization.

^†^ Calculated using the chi-square test.

^‡^ WHO Type II refers to the differentiated non-keratinizing carcinoma; WHO Type III refers to the undifferentiated non-keratinizing carcinoma.

^§^ According to the 8th edition of the AJCC/UICC Staging System.

^¶^ Others included patients with alteration of NAC regimens, for example switch from TPF to GP due to adverse events.

**Table S3: Comparison of baseline characteristics between cBR+SD/PD and non-cBR+SD/PD subgroups**

| **Characteristics** | **cBR+SD/PD**  **(N = 45)** | **Non-cBR+SD/PD**  **(N = 72)** | ***P^†^*** |
| --- | --- | --- | --- |
|  | **No. (%)** | **No. (%)** |  |
| Age, years |  |  | 0.32 |
| <45 | 30 (66.7) | 40 (55.6) |  |
| ≥45 | 15 (33.3) | 32 (44.4) |  |
| Sex |  |  | 0.52 |
| Male | 38 (84.4) | 56 (77.8) |  |
| Female | 7 (15.6) | 16 (22.2) |  |
| Histology, WHO type^‡^ |  |  | 1.00 |
| II | 0 (0.0) | 1 (1.4) |  |
| III | 45 (100.0) | 71 (98.6) |  |
| Smoking |  |  | 0.48 |
| No | 30 (66.7) | 42 (58.3) |  |
| Yes | 15 (33.3) | 30 (41.7) |  |
| Alcohol |  |  | 0.45 |
| No | 40 (88.9) | 59 (81.9) |  |
| Yes | 5 (11.1) | 13 (18.1) |  |
| Clinical stage^§^ |  |  | 0.82 |
| III | 18 (40.0) | 26 (36.1) |  |
| IV | 27 (60.0) | 46 (63.9) |  |
| T stage^§^ |  |  | 0.37 |
| T1 | 2 (4.4) | 5 (6.9) |  |
| T2 | 1 (2.2) | 7 (9.7) |  |
| T3 | 23 (51.1) | 30 (41.7) |  |
| T4 | 19 (42.2) | 30 (41.7) |  |
| N stage^§^ |  |  | 0.06 |
| N0 | 4 (8.9) | 3 (4.2) |  |
| N1 | 21 (46.7) | 21 (29.2) |  |
| N2 | 8 (17.8) | 28 (38.9) |  |
| N3 | 12 (26.7) | 20 (27.8) |  |
| Pre cfEBV DNA  (copies/mL) |  |  | <0.01 |
| <2 × 10^3^ | 17 (37.8) | 7 (9.7) |  |
| ≥2 × 10^3^ | 28 (62.2) | 65 (90.3) |  |
| NAC regimens |  |  | 0.86 |
| TPF | 1 (2.2) | 4 (5.6) |  |
| TP | 1 (2.2) | 1 (1.4) |  |
| GP | 6 (13.3) | 8 (11.1) |  |
| PF | 14 (31.1) | 26 (36.1) |  |
| Others^¶^ | 23 (51.1) | 33 (45.8) |  |
| NAC cycles |  |  | 0.16 |
| 2 cycles | 26 (57.8) | 42 (58.3) |  |
| 3 cycles | 14 (31.1) | 28 (38.9) |  |
| 4 cycles | 5 (11.1) | 2 (2.8) |  |

Abbreviations: cBR, complete biological response; cfEBV DNA, cell-free Epstein-Barr virus DNA; GP, gemcitabine and cisplatin; N, node; NAC, neoadjuvant chemotherapy; non-cBR, non-complete biological response; PF, cisplatin and 5-fluorouracil; PR, partial responses; T, tumor; TP, docetaxel and cisplatin; TPF, docetaxel, cisplatin, and 5-fluorouracil; WHO, World Health Organization.

^†^ Calculated using the chi-square test.

^‡^ WHO Type II refers to the differentiated non-keratinizing carcinoma; WHO Type III refers to the undifferentiated non-keratinizing carcinoma.

^§^ According to the 8th edition of the AJCC/UICC Staging System.

^¶^ Others included patients with alteration of NAC regimens, for example switch from TPF to GP due to adverse events.
